# Depression Partially Mediates the Association Between Binge Eating Disorder and Health-Related Quality of Life

**DOI:** 10.3389/fpsyg.2019.00209

**Published:** 2019-02-26

**Authors:** Christopher Singleton, Therese E. Kenny, Darcy Hallett, Jacqueline C. Carter

**Affiliations:** Department of Psychology, Memorial University of Newfoundland, St. John’s, NL, Canada

**Keywords:** binge eating disorder, health-related quality of life, depression, anxiety, eating disorders

## Abstract

Research has found that individuals with binge eating disorder (BED) report significantly worse health-related quality of life (HRQL) than those without eating disorders. Studies indicate that the association between BED and HRQL is largely accounted for by psychopathology (e.g., depression), rather than physiology [e.g., increased body mass index (BMI)]. However, to our knowledge, no study has yet investigated whether mental health symptoms could potentially mediate the relationship between BED and HRQL. To this aim, the present study compared a sample of adults who met DSM-5 criteria for BED (*n* = 72) recruited from the community for a treatment trial and a community sample of individuals with no history of an eating disorder (NED; *n* = 79). Participants completed self-report measures of HRQL (Short-Form 6D), eating disorder psychopathology (Eating Disorder Examination Questionnaire), and anxiety and depressive symptoms (Brief Symptom Inventory). Consistent with previous findings, the BED group reported significantly worse HRQL than the NED group after controlling for age, BMI, anxiety, depression, and eating disorder psychopathology. Moreover, depression partially mediated the relationship between BED diagnosis and HRQL. These results suggest that lessened HRQL may be partly explained by comorbid symptoms of depression in BED. Clinicians may find it helpful to specifically assess and treat depression in BED as a means of enhancing patients’ well-being. Future research should replicate these findings using longitudinal data that will allow for causal inferences.

## Introduction

Binge eating disorder (BED) is characterized by recurrent binge eating episodes, in which an individual eats an objectively large amount of food in a discrete period of time while experiencing a subjective sense of loss of control (American Psychiatric Association [APA], 2013). BED appears to have a significant, negative impact on the lives of individuals with the disorder. Specifically, BED has been associated with increased mental health concerns such as anxiety and depression (Hudson et al., 2007), physical health problems (Cremonini et al., 2009; Trace et al., 2012; Mitchell et al., 2015; Succurro et al., 2015), and functional impairments (Rieger et al., 2005), key facets of “health-related quality of life” (HRQL).

Health-related quality of life is a multidimensional construct that refers to an individual’s subjective appraisal of their health and the impact of their health on their day-to-day life (Ware et al., 1995; Centre for Disease Control, 2018). HRQL gauges areas such as physical and mental health, socializing and role functioning, pain, and vitality (Ware et al., 1995; Brazier and Roberts, 2004). A number of studies have found that those with BED report worse HRQL than control groups without BED (Masheb and Grilo, 2004; Rieger et al., 2005; Grenon et al., 2010; Ágh et al., 2015, 2016), prompting investigation into the specific health-related factors that influence the relationship between BED and poorer HRQL.

It has been argued that increased rates of obesity among individuals with BED (Hudson et al., 2007) may account for the association between BED and poorer HRQL (Costa and Pinto, 2015). However, while BED severity is positive correlated with obesity severity (Bruce and Agras, 1992; de Zwaan, 2001; Hudson et al., 2007), multiple studies have demonstrated that those with obesity and BED report significantly lower HRQL than those with obesity without BED, suggesting that the connection between BED and worse HRQL cannot be entirely accounted for by factors related to obesity (Hsu et al., 2002; Masheb and Grilo, 2004; Rieger et al., 2005; Perez and Warren, 2012; Vancampfort et al., 2014). For instance, in a sample of adults with obesity, those who endorsed BED symptomatology reported significantly worse HRQL, including social and role functioning, than those without BED symptoms (Hsu et al., 2002). Another cross-sectional study found that people with BED and obesity reported lower mobility, vitality, as well as social and role functioning than those with obesity, without BED (Vancampfort et al., 2014).

Consequently, research has turned to the role of psychological factors as a mechanism for lower HRQL in BED. For example, Rieger et al. (2005) reported that in a sample of individuals with obesity those with BED reported significantly worse weight-related self-esteem and public distress than those without BED. Similarly, Perez and Warren (2012) reported that while obesity was predictive of physical-related HRQL variables, BED diagnosis was more predictive of mental-health related HRQL variables. These findings align with research showing that BED is comorbid with other mental health conditions that may contribute to poorer HRQL, such as depression and anxiety (Hudson et al., 2007).

Given the particularly high comorbidity of BED and depression (Fontenelle et al., 2003; Bittencourt et al., 2012) studies have looked at the role of depression symptoms in the association between BED and HRQL. Evidence suggests that those with comorbid depression symptoms exhibited worse mental HRQL than those without depressive symptoms (Masheb and Grilo, 2004). Moreover, depressive symptoms predicted worse overall HRQL in BED after controlling for BMI and age (Grenon et al., 2010). Decreased energy, impaired concentration, and social isolation characteristic of depression may represent possible mechanisms through which depressive symptoms contribute to worsened HRQL (Trivedi et al., 2006). By extension, comorbid symptoms of depression may, at least partially, account for worse HRQL among individuals with BED.

To date, few studies have examined the specific role of comorbid depressive symptoms in the relationship between HRQL and BED. Most studies have used general mental health scales embedded within HRQL measures (Rieger et al., 2005; Perez and Warren, 2012). For example, Rieger et al. (2005) used the Impact of Weight on Quality of Life-Lite (IQWL; Kolotkin et al., 2001) mental health subscales (i.e., public distress, self-esteem) and Perez and Warren (2012) used an *ad hoc* HRQL scale with a single mental health-related item. Studies that have exampled depressive symptoms through non-HRQL measures in BED have not controlled for anxiety symptoms (Masheb and Grilo, 2004; Grenon et al., 2010) – a methodological limitation due to the substantial construct overlap between anxiety and depression (Endler and Macrodimitris, 2003; Löwe et al., 2008). Moreover, to our knowledge, there have been no formal cross-sectional or longitudinal mediation analyses investigating the mechanisms through which mental health difficulties in BED may contribute to worse HRQL. Thus, while findings suggest increased depressive symptoms and decreased HRQL among individuals with BED the *presence* and *nature* of this association remains unclear.

To address these gaps, the current study had two primary objectives. First, we aimed to examine HRQL in a sample of adults with BED recruited from the community for a treatment study and a community sample of individuals with no history of an eating disorder (NED). It was hypothesized that participants with BED would report significantly worse HRQL than those with NED, after controlling for body mass index (BMI), age, depression, anxiety, and eating disorder psychopathology. Given past findings suggesting a link between depression and HRQL in BED (Masheb and Grilo, 2004; Grenon et al., 2010), the second objective was to test whether comorbid depressive symptoms in BED mediated the relationship between BED diagnosis and HRQL while controlling for current anxiety symptoms.

## Materials and Methods

### Participants

A community sample of adults (19–65) with BED and adults with no history of an eating disorder (NED) were recruited directly from the community for a treatment study using various advertising initiatives (e.g., posters and brochures in community settings such as shopping malls and libraries; waiting rooms of local hospitals and medical clinics; bulletin boards around the local university and community college campuses; and advertisements on public radio and in local newspapers). No participants were recruited via referrals from clinical settings. The advertisements stated that researchers were interested in interviewing people “concerned about overeating” to determine if they might be eligible to take part in a study evaluating a new self-help program. Only baseline data for the BED participants were examined in the current study.

### BED Group

Individuals were eligible for the BED group (and treatment trial assessing the efficacy of dialectical behavior therapy self-help for BED) if they met DSM-5 criteria for BED as determined by the Eating Disorder Examination (EDE) 17.0 semi-structured interview (Fairburn et al., 2014). Other inclusion criteria for the trial were: age between 19 and 65; BMI of ≥18.5 kg/m^2^; private access to device with a microphone, camera, and Internet connection; and proficiency with reading and writing in English. The latter two criteria were necessary for participation in the treatment trial. Exclusion criteria included current involvement in treatment for binge eating with a registered psychologist, a major medical illness that might impact eating or weight (e.g., diabetes), acute suicidal ideation, and potential substance use issues (i.e., a score of five or greater on the Drug Abuse Screening Test [Skinner, 1982] or 16 or greater on the Alcohol Use Disorders Identification Test; [Saunders et al., 1993]). Individuals taking antidepressant or sleep medication were eligible if they reported no change in dosage over the past 3 months.

### NED Group

Adults (19–65) who did not self-report a past or present eating disorder (ED) diagnosis were eligible to participate in the NED group. The SCOFF questionnaire (Morgan et al., 1999) was administered to identify participants who may have exhibited ED symptomatology. The SCOFF questionnaire includes five yes-or-no questions and is designed to identify individuals at risk of an eating disorder. In the current study, those who reported a score of three or higher (Hill et al., 2010) were not eligible to take part in the NED group and were sent local eating disorder resources. All other inclusion and exclusion criteria remained identical to the BED group to increase the likelihood of the groups being as similar as possible.

### Measures

#### BED Diagnosis

BED diagnosis according to DSM-5 was determined through the EDE 17.0 (Fairburn et al., 2014) via telephone interview. The EDE 17.0 is a semi-structured diagnostic interview that assesses the presence and severity of ED symptoms. Numerous studies suggest that the EDE possesses strong psychometric properties (see Berg et al., 2012). In order to minimize participant burden, only the BED diagnostic module and questions used to rule out extreme compensatory behaviors (i.e., self-induced vomiting, laxative/diuretic misuse, excessive exercise, avoidance of eating) were administered. Prospective participants were deemed to meet BED criteria if they reported at least 12 objective binge episodes within the past 3 months in the absence regular extreme compensatory behaviors (i.e., conservatively defined as fewer than six episodes over the past 6 months). In addition, a diagnosis of BED also required three of the following five binge characteristics: (i) eating more rapidly than normal; (ii) eating until comfortably full; (iii) eating large amounts of food when not physically hungry; (iv) eating alone due to embarrassment toward amount of food being consumed; (v) feeling disgusted with oneself, ashamed, or guilty following binge episode. Finally, endorsement of distress and/or impairment related to binge eating was required (American Psychiatric Association [APA], 2013).

Trained graduate students conducted the EDE interviews under the supervision of the senior author (JC). As previous studies have indicated that the EDE has good inter-rater reliability (e.g., Rizvi et al., 2000), inter-rater reliability was not assessed in the current study. Moreover, interview data that was difficult to rate (e.g., determining whether a binge episode was objectively large) were discussed among the research team until a consensus was reached. Interviewers received weekly supervision and were evaluated by the primary investigator (JC) during the study to ensure standardization and quality of EDE administration.

#### ED Psychopathology

The Eating Disorder Examination Questionnaire (EDE-Q) 6.0 (Fairburn and Beglin, 2008) was used to measure global ED psychopathology. The EDE-Q is a 28-item self-report version of the EDE interview (Fairburn et al., 2014) and uses a seven-point Likert scale, with higher scores reflecting more severe ED symptoms. The present study used a seven-item, three-factor (dietary restraint, overvaluation of weight and shape, and body dissatisfaction) version of the EDE-Q, as this version has been validated in the general population (Grilo et al., 2015), as well as patients with obesity (Grilo et al., 2013), and BED (Grilo et al., 2010). The current analysis only employed the EDE-Q global score, an indicator of overall ED symptom severity. Internal consistency for the EDE-Q was good (Cronbach’s alpha = 0.84 for the Global scale).

#### HRQL

The Short Form 6D (SF-6D) was administered in order to gauge participants’ HRQL. The SF-6D is derived from the widely used and validated SF-12. In developing this measure, the researchers had a large United Kingdom sample (*n* = 611) rank their preferred SF-12 score profile (i.e., combination of scores on the SF-12 scales assessing HRQL domains of physical health, mental health, socializing, role functioning, pain, and vitality). The preferences indicated in this study were used to develop index scores that indicate an individual’s “health state” relative to the preferences indicated in the general population. Scores range from 0.296 (significant challenges in all domains) to 1 (no problems in any domain). The SF-6D has demonstrated strong construct, convergent, and discriminant validity in its use with mental health issues such as depression and anxiety (Lamers et al., 2006; Mulhern et al., 2014). The SF-6D showed good internal consistency in the present study (Cronbach’s alpha = 0.83).

#### Depression and Anxiety

Depressive and anxious symptoms were assessed using the Brief Symptom Inventory (BSI; Derogatis and Melisaratos, 1983), a 53-item, psychometrically strong measure that assesses psychological distress over the past week. Individuals are prompted to rate responses on a five-point Likert scale, with higher scores indicating more severe psychological symptoms. The current study used the BSI Depression and Anxiety scales. While the original BSI assesses distress in the past week, we changed the time frame to the past month so that this measure corresponded to the eating disorder psychopathology measure used in the larger study (Fairburn, 2008). Internal consistency in the current study was good for the Depression (Cronbach’s alpha = 0.88) and Anxiety (Cronbach’s alpha = 0.87) subscales.

### Procedure

Individuals who responded to study advertisements were directed to an online screening questionnaire. Those meeting initial eligibility criteria for the BED group were contacted to schedule a telephone interview to confirm BED diagnosis. Eligible participants were sent an information form for the treatment study and a link to the study measures and demographic questionnaire. Similarly, following completion of the screening questionnaire, those eligible for the NED group were sent a link to the information form for the study questionnaires, and demographic form. Individuals in the NED group were entered into a draw for a $100 gift card following completion of the study questionnaires.

With regards to participation, the present study employed an implied consent model. The information forms for both groups (presented prior to the questionnaires) thoroughly reviewed consent-related matters (e.g., risks and benefits). Participant consent was implied with completion of the questionnaires, as all participants completed the study via distance. The present study was conducted in line with provincial safety and ethical standards, and was approved by the Newfoundland and Labrador Health Research Ethics Board.

### Data Analysis

Data analysis was conducted using IBM SPSS Software (Armonk, NY, United States), with α = 0.05. Prior to running the analyses, data were tested for normality and equality of variances. All variables demonstrated homogeneous variance. HRQL was transformed via a logarithmic transformation to produce a normal distribution.

First, differences between the BED and NED groups in terms of demographic and clinical variables were examined using independent samples *t*-tests for continuous variables and Chi-square tests of independence for categorical variables. To correct for multiple comparisons, a Bonferroni correction of (0.05/6 = 0.008) was applied.

To address the first research question, we employed an ANCOVA with group (BED or NED) as the independent variable and SF-6D score as the dependent variable. Demographic and clinical variables that emerged as significant in the between-group comparisons (i.e., BMI, age, depression, anxiety, and eating disorder psychopathology) were entered as covariates.

Before examining the second research question, we wanted to ensure that group differences in BSI anxiety could not account for group differences in BSI depression (Endler and Macrodimitris, 2003; Löwe et al., 2008). Therefore, we conducted two linear regressions with group as the predictor, either BSI anxiety or depression as the criterion, and with the other BSI variable held constant. Having demonstrated that the association between depressive symptoms and BED diagnosis could not be accounted for by anxiety symptoms, a mediation analysis was conducted according to Baron and Kenny (1986) guidelines, with BSI anxiety score held constant. As in the ANCOVA, variables that were significantly different between the BED and NED groups (i.e., BMI, age, depression, anxiety, and eating disorder psychopathology) were controlled in this analysis. Of note, given the cross-sectional nature of the data, the present mediation analysis was an *atemporal* mediation, yielding correlational results (i.e., as opposed to a *temporal* mediation, which requires longitudinal data and which may indicate causal findings; Winer et al., 2016). First, a linear regression was conducted with control variables in the first block, group as the predictor in the second block, and BSI depression as the criterion. Next, we conducted a linear regression with control variables in the first block, group as the predictor, and SF-6D score as the criterion. Lastly, a linear regression was run with control variables and BSI depression in the first block, followed by group in the second block, and SF-6D score as the criterion. A *post hoc* Sobel test was conducted to determine whether the decrease in the regression coefficient for group suggested a significant atemporal mediation.

## Results

### Sample Characteristics

Of the individuals who completed the study questionnaires, six participants from the NED group and one participant from the BED group were retroactively excluded from the present analyses due to a reported BMI less than 18.5 kg/m^2^ (*n* = 4), hypothyroidism (*n* = 2), or type II diabetes (*n* = 1). The final sample therefore consisted of 72 individuals in the BED group (93.1% female; *n* = 67) and 79 participants in the NED group (83.5% female; *n* = 66).

Participant and clinical characteristics are presented in Table 1. Independent samples *t*-test revealed that the BED group reported significantly higher BMI (large effect size) and age (medium effect size) than the NED group. There were no other significant between-group differences in demographic variables. Consistent with previous research, the BED group reported higher BSI Depression and EDE-Q Global scores than the NED group. They also reported worse HRQL than in comparison to the NED group. Although groups differed on BSI Anxiety at the 0.05 level, this finding was non-significant at the corrected 0.008 level.

**Table 1:** Participant characteristics for the NED and BED groups.

	Group				
	NED (*n* = 79) Mean (*SD*)	BED (n = 72) Mean (*SD*)	*t*	*p*	Cohen’s *d*
**Participant characteristics**					
BMI	26.56 (5.80)	37.55 (9.57)	-0.86	<0.01	1.38
Age	33.87 (11.45)	40.56 (11.45)	-3.24	<0.01	0.55
**Clinical characteristics**					
EDE-Q global	2.66 (1.55)	4.46 (0.93)	-8.76	<0.01	1.41
BSI					
Anxiety	0.78 (0.079)	1.12 (0.81)	-2.65	0.01	0.42
Depression	0.87 (0.084)	1.34 (0.79)	-3.60	<0.01	0.59
SF-6D	0.74 (0.12)	0.64 (0.11)	5.66	<0.01	0.87


*A Bonferroni correction was used where significance was set at p < 0.008.*

### Between-Group HRQL Differences

An ANCOVA was run to determine whether groups significantly differed on SF-6D scores after controlling for age, BMI, BSI Anxiety, and BSI Depression, and EDE-Q Global score. Results indicated that the BED group reported significantly worse HRQL compared to the NED group after accounting for covariates, *F*(1,150) = 7.485, *p* = 0.007, $\eta_{p}^{2}$ = 0.05 (small effect size). Moreover, BSI Anxiety, *F*(1,150) = 5.700, *p* = 0.018, $\eta_{p}^{2}$ = 0.04 (small effect size), and BSI Depression, *F*(1,150) = 17.475, *p* < 0.001, $\eta_{p}^{2}$ = 0.11 (small effect size), accounted for a significant portion of the between-group variance in SF-6D scores. BMI, *F*(1,150) = 2.328, *p* = 0.129, age, *F*(1,150) = 0.075, *p* = 0.785, and Global EDE-Q score, *F*(1,150) = 0.199, *p* = 0.656, accounted for significant variance in HRQL.

### Mediation Analysis

Prior to running a mediation analysis, two linear regression analyses were conducted to investigate potential construct overlap in BSI Depression and BSI Anxiety. The analyses revealed that group significantly predicted BSI Depression when BSI Anxiety was held constant, β = 0.208, *SE* = 0.087, *t*(1) = 2.379, *p* = 0.019, but that group did not predict BSI Anxiety when BSI Depression was held constant, β = -0.016, *SE* = 0.086, *t*(1) = -0.181, *p* = 0.857, suggesting that depression accounts for the between-group variance in BSI Anxiety. As such, we next investigated whether BSI Depression mediated the relationship between group and SF-6D score, while holding BSI Anxiety constant. While previous studies have suggested that obesity may account for the association between BED diagnosis and HRQL, BMI was not significantly associated with HRQL in the ANCOVA analysis and was therefore not included in the atemporal mediation model.

**FIGURE 1:**
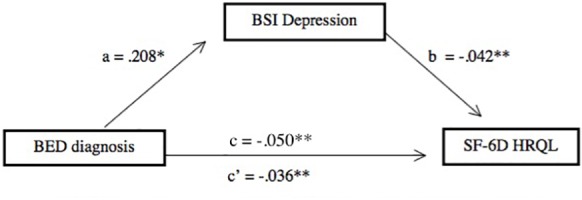
Brief Symptom Inventory Depression score partially mediated the association between BED diagnosis and HRQL after controlling for BSI Anxiety. ^∗^*p* < 0.05; ^∗∗^*p* < 0.001.

The first regression demonstrated that group significantly predicted SF-6D score while controlling for BSI Anxiety, β = -0.050, *SE* = 0.010, *t*(1) = -4.87, *p* < 0.001, Δ*R*^2^ = 0.087. As reported above, group also significantly predicted BSI Depression score while controlling for BSI Anxiety. Having thus satisfied the first two conditions of Baron and Kenny (1986) model, we next ran a linear regression with SF-6D score as the criterion, BSI Anxiety and BSI Depression in the first block, and Group in the second block. Both BSI Depression, β = -0.042, *SE* = 0.009, *t*(3) = -4.407, *p* < 0.001, Δ*R*^2^ = 0.139, and group, β = -0.036, *SE* = 0.01, *t*(3) = -4.227, *p* < 0.001, Δ*R*^2^ = 0.057, significantly predicted SF-6D scores. However, it was noted that the association between BED diagnosis and HRQL decreased after controlling for BSI Depression. A *post hoc* Sobel test revealed that this decrease was significant, *z* = -2.106, *p* = 0.04, suggesting that depressive symptoms partially mediated the association between group and SF-6D score (see Figure 1).

## Discussion

The present study aimed to compare self-reported HRQL of adults with BED to a control group with no history of an eating disorder (NED) and to investigate whether depression symptoms mediate the relationship between BED diagnosis and HRQL. Our first main finding was that the BED group reported significantly lower HRQL than the NED group after controlling for relevant covariates, consistent with our hypothesis. This has been repeatedly demonstrated in the literature (Masheb and Grilo, 2004; Rieger et al., 2005; Grenon et al., 2010; Ágh et al., 2015, 2016), suggesting that worse HRQL is a cardinal feature of BED. The current finding is particularly noteworthy as this is the first paper to replicate this association in a community sample (who responded to an advertisement for a treatment study), as opposed to a sample recruited from a clinical setting. In addition, the between-group difference in HRQL remained after controlling for BMI and age. Further, BMI was not significantly associated with HRQL in the ANCOVA analysis. This finding adds to accumulating evidence suggesting that the relationship between BED and HRQL is not entirely accounted for by weight status (Masheb and Grilo, 2004; Grenon et al., 2010).

In addition to BED diagnostic status, BSI Depression shared a significant relation with HRQL. An atemporal mediation analysis revealed that depression symptoms account for some of the variance in the relationship between BED diagnosis and HRQL, after controlling for overlapping variance with the anxiety construct. This finding aligns with prior longitudinal research findings suggesting an association between depression and HRQL within individuals with BED (Masheb and Grilo, 2004; Rieger et al., 2005; Grenon et al., 2010; Perez and Warren, 2012). This pattern has been documented in bariatric surgery studies indicating coinciding improvement in mood, HRQL, and eating disorder symptoms following surgical intervention among those with obesity and BED (Faulconbridge et al., 2013; Mack et al., 2016). Furthermore, the present results support these findings by suggesting that depression may be a specific mechanism through which HRQL is worsened in BED.

Taken together, the present findings may have important clinical implications. Not only does decreased HRQL appear to be a cardinal feature of BED, but it also seems that co-occurring depression symptoms may contribute to this phenomenon. Depression symptoms have been found to worsen HRQL by impairing areas such as energy and social functioning (Trivedi et al., 2006). Thus, our results, although correlational, indicate that clinicians may find it beneficial to assess for comorbid depression symptoms and address them in BED treatment. Specifically, integrating behavioral activation components into BED treatment may help both assuage co-occurring depression symptoms and subsequently improve HRQL (e.g., augmenting activity levels, socializing, and role functioning; Veale, 2008; Beck, 2011

In concert with clinical considerations, the current results align with empirical findings that point toward similarities between BED and other eating disorders. Specifically, prior research has shown that individuals with anorexia nervosa (AN) and bulimia nervosa (BN) also report decreased HRQL (Fox and Leung, 2009; Pohjolainen et al., 2010) and these detriments are associated with comorbid psychopathology in AN and BN (e.g., depression; Padierna et al., 2000; González-Pinto et al., 2004; Ágh et al., 2016). This suggests that HRQL may be similarly affected across the other forms of eating disorders, despite differences in symptom presentation, consistent with a transdiagnostic model (Fairburn et al., 2003). Thus BED may share a more similar HRQL profile with other eating disorders than obesity, despite the increased prevalence of obesity in BED (de Zwaan, 2001; Hudson et al., 2007). This is consistent with research suggesting that obesity impacts HRQL mainly through physiological complications (Fontaine and Barofsky, 2001; Jia and Lubetkin, 2005) while HRQL seems to be impacted by psychological factors in BED (Masheb and Grilo, 2004; Rieger et al., 2005; Grenon et al., 2010; Perez and Warren, 2012). Indeed, the current study found no association between BMI and HRQL. Research in this area is preliminary, however, and further studies are needed to shed light on this question.

It is important to note that the current study had limitations. First, since this was a correlational cross-sectional study, we cannot say definitively that BED causes worse HRQL or that depression is directly linked to poor HRQL in BED. Nor are we able to draw conclusions about the direction or temporal relationship between these variables. Future studies should replicate the present analyses with longitudinal data that may allow for causal conclusions. In addition, the BED and NED groups were not matched on BMI. It is possible, given the association between obesity and poor HRQL (Costa and Pinto, 2015) that BMI could have accounted for HRQL differences between diagnostic groups. BMI, however, did not contribute unique variance to HRQL in the current results.

With regards to the BED assessment, while the EDE has demonstrated strong inter-rater reliability (e.g., Berg et al., 2012) and the interview was administered by trained assessors who were closely supervised, we did not assess for inter-rater reliability. Therefore, we cannot comment on the reliability of this measure in the current study. Furthermore, the NED group did not complete the EDE interview and instead completed self-report screening measures. This may have resulted in inaccurate reporting of BED symptoms in the NED group. The difference in EDE-Q Global score, however, suggests the validity of these groupings. Moreover, EDE-Q scores in the NED sample were comparable to a community sample using the same modified three-factor, seven-item structure (Grilo et al., 2015).

These limitations notwithstanding, this study also had a number of methodological strengths. Our use of a sample recruited directly from the community may increase the external validity of our results. Moreover, the mediation analysis – although atemporal – provides new evidence on the nature of the relationship between HRQL and BED, though we acknowledge that this analysis was conducted with cross-sectional data and is thus subject to interpretive limitations as discussed above. Lastly, unlike previous studies that linked depression symptoms with diminished HRQL in BED, we controlled for anxiety symptoms in our analyses. As depression and anxiety have been found to have substantial construct overlap (Endler and Macrodimitris, 2003; Zbozinek et al., 2012), this procedure helped us distinguish the role of these two closely related variables, and increases confidence that specific symptoms unique to depression contributed to the worse HRQL in the BED group.

Taken together, we have replicated the finding that those with BED experience worse HRQL than those without BED after adjusting for BMI and age. In addition, we have demonstrated that depression symptoms may partially mediate this relationship, supporting previous research that highlighting the role of psychopathology, as opposed to physical health issues, in explaining this relationship. As depression only partially mediated this relationship, future research should explore other potential contributors to worsened HRQL in BED, as an enhanced understanding of this relationship could have substantial clinical implications. In addition, longitudinal research is needed in order to confirm the temporal nature of these relationships.

## Author Contributions

CS, TK, and JC helped to design the larger study and formulate the present research question. CS analyzed the data, wrote the manuscript, and designed the tables and figure. TK, DH, and JC provided subsequent feedback and contribution to the manuscript prior to submission. DH assisted with data analysis.

## Conflict of Interest Statement

The authors declare that the research was conducted in the absence of any commercial or financial relationships that could be construed as a potential conflict of interest.
